# Deep-learning-based enhanced optic-disc photography

**DOI:** 10.1371/journal.pone.0239913

**Published:** 2020-10-01

**Authors:** Ahnul Ha, Sukkyu Sun, Young Kook Kim, Jinho Lee, Jin Wook Jeoung, Hee Chan Kim, Ki Ho Park

**Affiliations:** 1 Department of Ophthalmology, Seoul National University College of Medicine, Seoul, Korea; 2 Department of Ophthalmology, Jeju National University Hospital, Jeju-si, Korea; 3 Interdisciplinary Program, Bioengineering Major, Graduate School, Seoul National University, Seoul, Korea; 4 Department of Ophthalmology, Seoul National University Hospital, Seoul, Korea; 5 Seoul Glaucoma Image Post-processing Laboratory (SGIP LAB), Seoul, Korea; 6 Department of Ophthalmology, Hallym University Chuncheon Sacred Heart Hospital, Chuncheon, Korea; 7 Department of Biomedical Engineering, Medical Research Center, Seoul National University College of Medicine, Seoul, Korea; Icahn School of Medicine at Mount Sinai, UNITED STATES

## Abstract

Optic-disc photography (ODP) has proven to be very useful for optic nerve evaluation in glaucoma. In real clinical practice, however, limited patient cooperation, small pupils, or media opacities can limit the performance of ODP. The purpose of this study was to propose a deep-learning approach for increased resolution and improved legibility of ODP by contrast, color, and brightness compensation. Each high-resolution original ODP was transformed into two counterparts: (1) down-scaled ‘low-resolution ODPs’, and (2) ‘compensated high-resolution ODPs’ produced via enhancement of the visibility of the optic disc margin and surrounding retinal vessels using a customized image post-processing algorithm. Then, the differences between these two counterparts were directly learned through a super-resolution generative adversarial network (SR-GAN). Finally, by inputting the high-resolution ODPs into SR-GAN, 4-times-up-scaled and overall-color-and-brightness-transformed ‘enhanced ODPs’ could be obtained. General ophthalmologists were instructed (1) to assess each ODP’s image quality, and (2) to note any abnormal findings, at 1-month intervals. The image quality score for the enhanced ODPs was significantly higher than that for the original ODP, and the overall optic disc hemorrhage (DH)-detection accuracy was significantly higher with the enhanced ODPs. We expect that this novel deep-learning approach will be applied to various types of ophthalmic images.

## Introduction

Optic nerve head (ONH) examination is essential to glaucoma diagnosis and progression assessment [1, 2]. Optic-disc photography (ODP) has been proven to be very effective for documentation of optic nerve appearance, as it allows for more detailed scrutinization and subsequent comparison for determination of progressive change [3–5]. Furthermore, ODP enables clinicians to qualitatively assess ONH structures such as detailed neuroretinal rim contours, presence of optic disc hemorrhage (DH), parapapillary chorioretinal atrophy (PPA) or vessel alterations, which is not possible in optical coherence tomography (OCT) [6].

In real clinical practice, limited patient cooperation, small pupils, or media opacities can limit the performance of ODP [7]. As a result, ODPs can have several limitations, such as insufficient resolution, low color contrast, and inconsistency of image quality (especially in cases of media opacity due to cataracts). Even when high-resolution ODPs can be obtained, red-colored blood vessels and red-orange-colored retina sometimes cause indistinct pathologies such as small-sized DH to be missed. ODP-quality improvement techniques that can obviate the limitations of the current imaging acquisition devices are essential, especially when considering the indispensability of ONH structural evaluation in glaucoma treatment.

The popularity of deep-learning algorithms offering modeling of high-level abstractions in data by means of multiple processing layers has exploded in recent years as powerful graphics processing units (GPUs) have become available. The very intricate process of high-resolution image estimation from a low-resolution counterpart is known as super-resolution (SR) [8, 9]. For image SR, generative adversarial network (GAN), which is a deep neural net architecture comprising two nets one pitted against the other (hence “adversarial”), has shown great utility and potential [10].

In this paper, we propose a modified super-resolution generative adversarial network (SR-GAN) that is capable not only of up-scaling but also of improving ODPs’ details as well as the visibility of the optic disc margin and surrounding retinal vessels in computing ‘enhanced’ ODPs. In the present study, we performed a quantitative evaluation to assess enhanced ODPs’ clinical utility for ONH evaluation.

## Methods

This study was approved by the Seoul National University Hospital Institutional Review Board (1805-027-944) and faithfully adhered to the tenets of the Declaration of Helsinki. All of the subjects provided their written informed consent. Eyes were chosen from subjects examined for glaucoma at the Glaucoma Clinic, Seoul National University Hospital, between January and December 2018. All of the relevant data are in the manuscript and its Supporting Information files.

### Design of generative adversarial network

**Fig 1:**
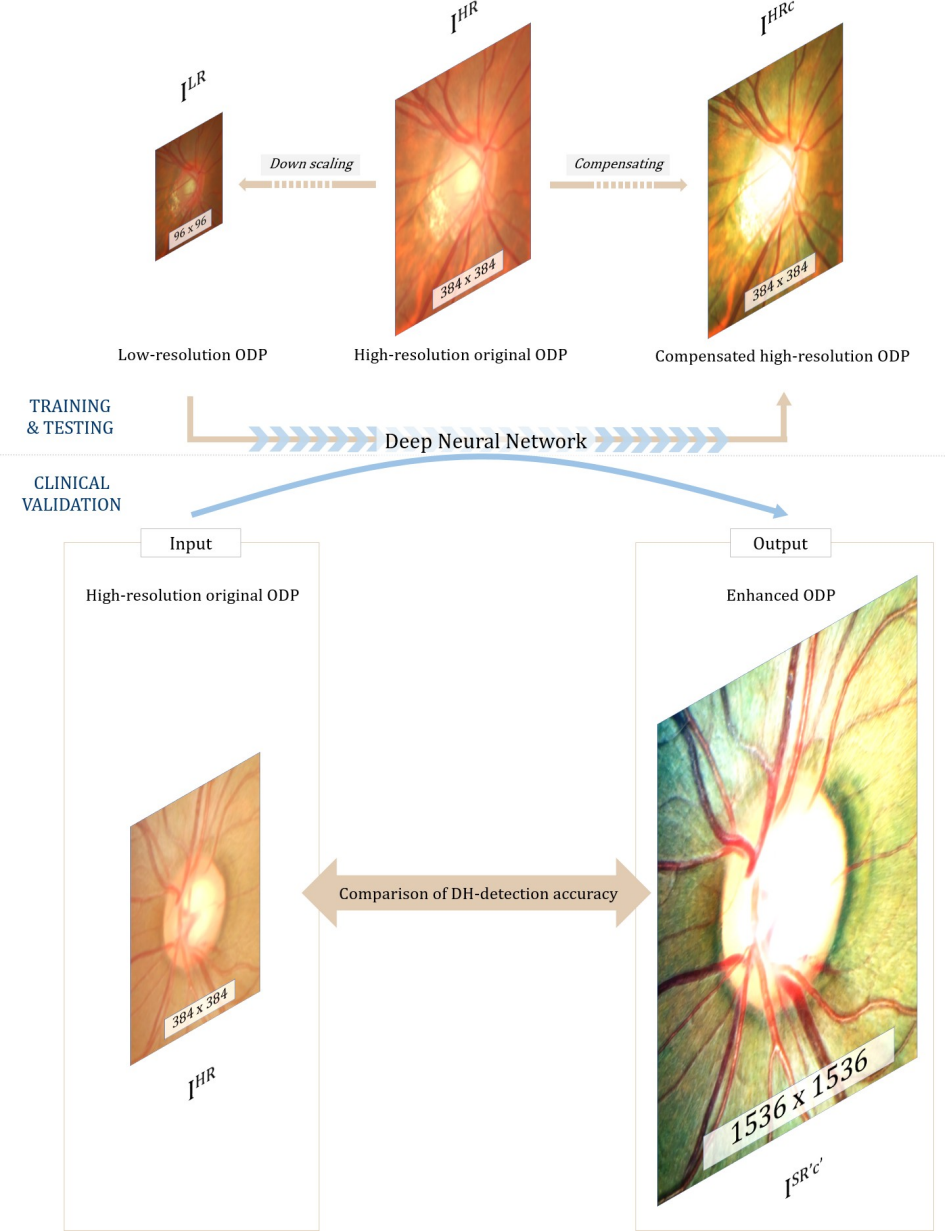
Principle of enhanced image formation via super-resolution generative adversarial network (SR-GAN). A modified SR-GAN was used to learn the differences between the low-resolution optic-disc photography (ODP) and the manually compensated high-resolution ODP. By inputting the high-resolution original ODP into the algorithm, an X4 up-scaled and overall contrast-, color- and brightness-transformed ‘enhanced ODP’ could be obtained.

An I^SR'c'^ is a super-resolved, compensated image, and an I^HR^ is the high-resolution original image. The final goal of this study was to obtain I^SR'c'^ (1536 x 1536 pixels) from an I^HR^ (384 x 384 pixels) for clinical validation (see Fig 1). For that purpose, the differences between the I^HRc^ (384 x 384 pixels) and I^LR^ (96 x 96 pixels) were learned directly through a modified SR-GAN. An I^LR^, which is the low-resolution version of the I^HR^, was obtained by 1/4 resizing of the I^HR^ using bicubic interpolation (down-scaled width and height: 1/4W x 1/4H x C) [11], the I^HRc^ being the high-resolution image manually customized by a post-processing algorithm. The SR-GAN consists of a GAN and a pre-trained VGG19 (Visual Geometry Group) network [12].

**Fig 2:**
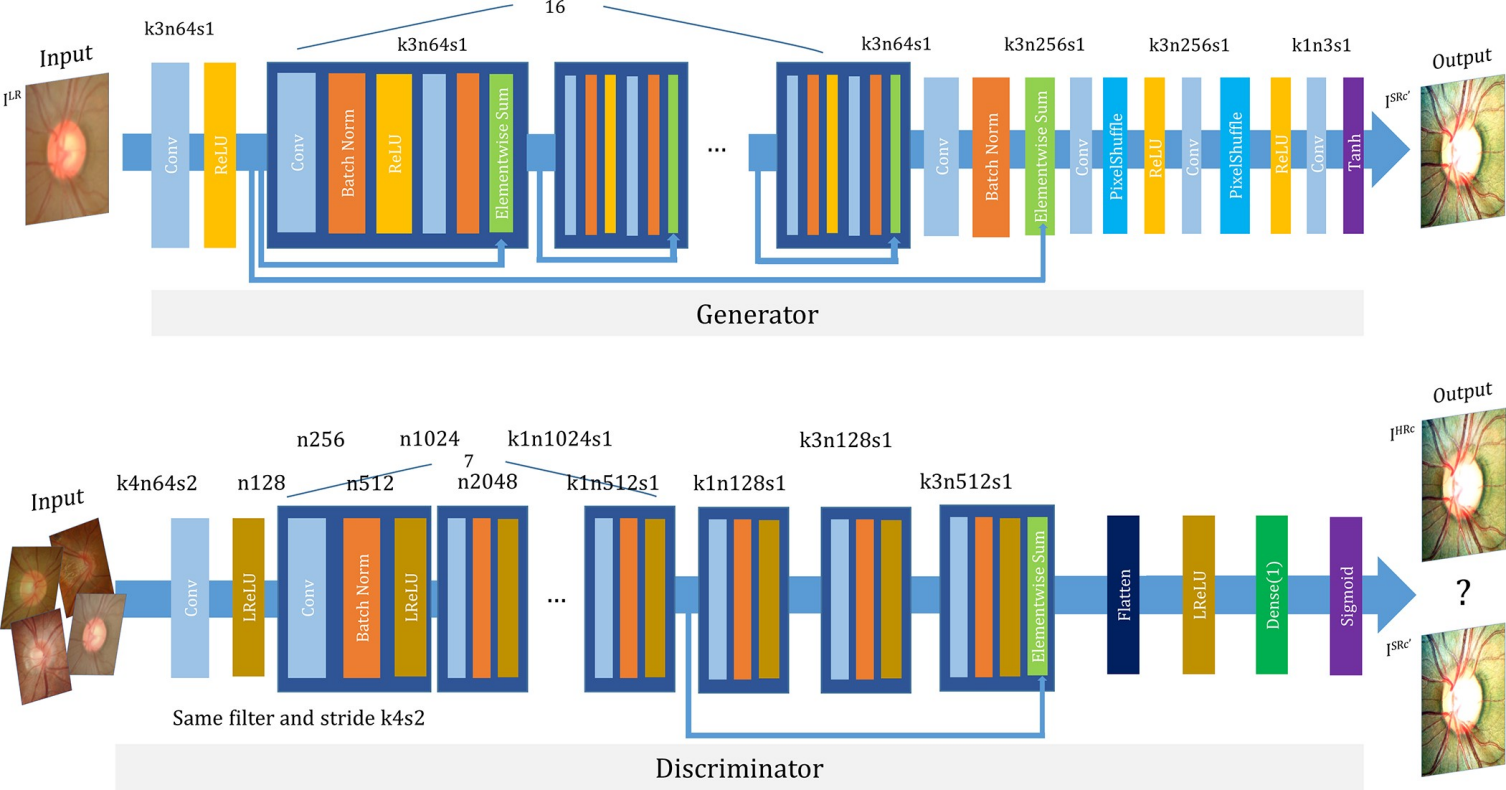
Architecture of generator and discriminator network. The corresponding kernel size (k), number of feature maps (n) and stride (s) are indicated for each convolutional layer.

The GAN includes an additional discriminator for evaluation of the generator’s reliability [13]. The discriminator makes a judgement on whether a randomly inputted image is a guess of the generator or a high-resolution measurement. For optimized discriminator judgement, an adversarial loss is created that iteratively optimizes the discriminator for enhanced decision-making capability. Also, the adversarial loss, together with the content loss, are used to optimize the generator in pushing it in the direction in which more perceptually realistic outputs can be generated to further fool the discriminator [10]. By this process of adversarial training, the quality of images from the generator can be improved. The training is terminated once the generator produces results that the discriminator cannot distinguish from the high-resolution images [14]. The generator and discriminator network architecture with the corresponding kernel size (k), number of feature maps (n) and stride (s) is shown in Fig 2. We applied Tensorlayer SubpixelConv2d as a PixelShuffle [15].

Each original ODP (I^HR^) was transformed into two counterparts: (1) down-scaled ‘low-resolution ODPs (I^LR^, 96 x 96 pixels)’ and (2) ‘compensated high-resolution ODPs (I^HRc^, 384 x 384 pixels)’ produced via enhancement of the visibility of the optic disc margin and surrounding retinal vessels using a customized image post-processing algorithm. Then, the differences between the two were directly learned through the modified SR-GAN. Finally, by inputting of the high-resolution original ODPs (I^HR^, 384 x 384 pixels) into the trained SR-GAN, 4-times-up-scaled and overall contrast-, color-, and brightness-transformed ‘enhanced ODPs (I^SR'c'^, 1536 x 1536 pixels)’ could be obtained.

### Loss functions

Our ultimate goal was to train a generating function G by training a generator network as a feed-forward CNN $G_{\theta_{G}}$ parametrized by *θ*_*G*_. Here, *θ*_*G*_ denotes the weight and bias of the designed network, and is obtained by optimizing loss function *l*^*SR*^. The sum of loss functions, *l*^*SR*^, is obtained. For training of image $I_{n}^{Obtained}$ with corresponding $I_{n}^{Target}$ n = 1, 2, 3 ⋯N, the following equation is solved:

$$\hat{\theta_{G}}{=}_{\theta_{G}}^{argmin}\frac{1}{N}\sum_{n=1}^{N}l^{SR}(G_{\theta_{G}}(I_{n}^{Obtained}),I_{n}^{Target})$$ (1)

First, the pixel-wise Mean Squared Error (MSE) loss was calculated as follows:

$$L_{MSE}=\frac{1}{r^{2}WH}\sum_{x=1}^{W_{i,j}}\sum_{y=1}^{H_{i,j}}{\left(I_{x,y}^{Target}-G_{\theta_{G}}{\left(I^{Obtained}\right)}_{x,y}\right)}^{2}$$ (2)

where *Wi*,*j*, *Hi*,*j* are the width and height, respectively, of the feature map. MSE loss, widely utilized for image SR, calculates the squared difference in pixels between the obtained and target images (the latter being a manually customized high-resolution image) during the training process. However, MSE optimization results in blurring of the edges of the generated image.

Therefore, we also adopted VGG loss as defined with the pre-trained (trained with ImageNet) VGG19 Network. VGG loss was calculated as follows:

$$L_{VGG/i.j}=\frac{1}{W_{i,j}H_{i,j}}\sum_{x=1}^{W_{i,j}}\sum_{y=1}^{H_{i,j}}({\emptyset_{i,j}(I^{Obtained})}_{x,y}-{{\emptyset_{i,j}(G_{\theta_{G}}(I^{Target}))}_{x,y})}^{2}$$ (3)

where *Øi*,*j* indicates the feature maps of the pre-trained VGG19 Network after the j^th^ convolution and before the i^th^ maxpooling layer. In this study, we employed feature maps of conv4_3 (j = 4, i = 3, *Wi*,*j* = 28, *Hi*,*j* = 28). VGG loss was used to calculate the squared difference between the feature maps of the target and generated images via SR-GAN. By using both MSE and VGG loss, the overall resolution and style of the generated image could be improved [16].

Finally, the adversarial loss function computes the Sigmoid Cross Entropy (SCE) loss by calculating the difference between the output logits of the generated image ($G_{\theta_{G}}(I^{LR})))$ and target image to fool the discriminator, as follows:

$$L_{Gen}=\sum_{n=1}^{N}-\log D_{\theta_{D}}(G_{\theta_{G}}(I^{LR}))$$ (4)

where $D_{\theta_{D}}(G_{\theta_{G}}(I^{LR})$ is the probability of ($G_{\theta_{G}}(I^{LR})))$ to be considered as a target image. The generator tries to fool the discriminator by generating higher-quality images. The final goal of adversarial loss is the minimization of $-\log\;D_{\theta_{D}}(G_{\theta_{G}}(I^{LR}))$.

### Dataset

ODPs were obtained post-pupil-dilation using a digital fundus camera system (CF‐60UVi/D60; Canon, Inc., Tokyo, Japan). The images were saved in the 384 x 384-pixel digital imaging and communications in medicine format and stored in the picture archiving communication system (PACS) of Seoul National University Hospital.

### Details on customized image post-processing algorithm

**Fig 3:**
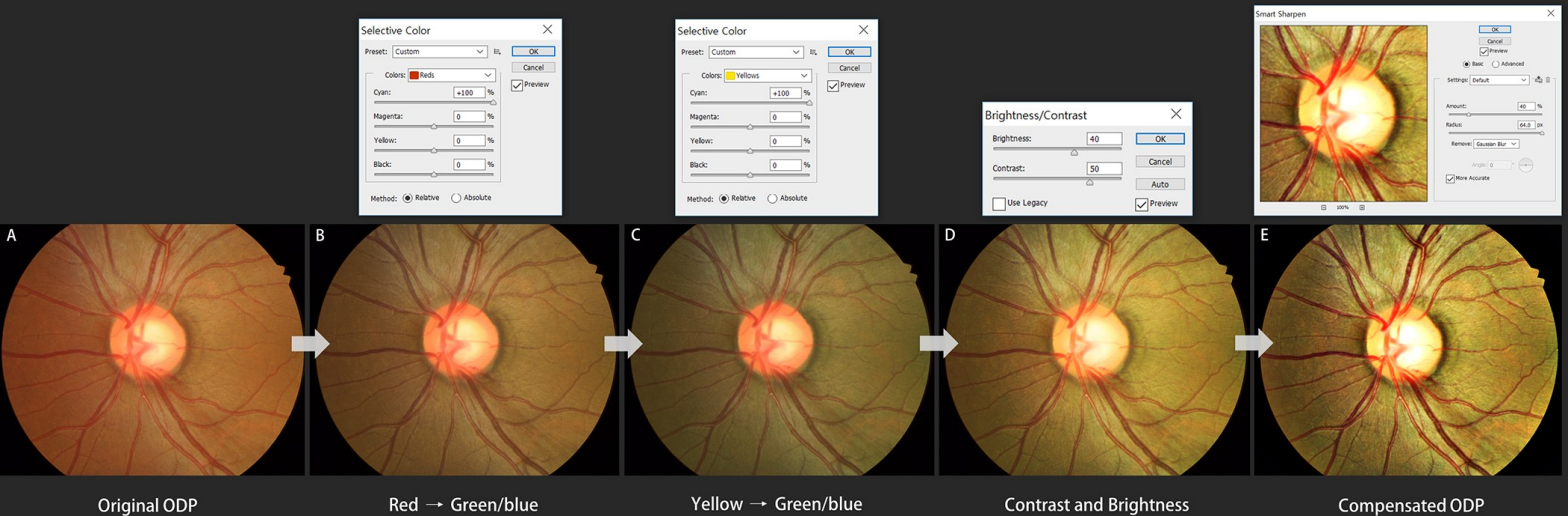
Customized image post-processing algorithm for maximized visibility of hemorrhage. (A) Original ODP. (B) With Selective color tool, red color was replaced by green/blue and (C) yellow was replaced by green/blue. (D) With the Contrast/Brightness tool, the contrast level was improved, and with the Smarten sharpen tool, the degree and range of the sharpness was increased. (E) Finally, a compensated high-resolution ODP could be obtained.

The purpose of this type of processing is to generate an improved ODP image in terms of both color and spatial contrast. The processing entails the following steps: contrast optimization, edge enhancement, spatial and frequency filtering, image combining, and noise reduction. Detailed manual adjustment has to be applied differently according to each ODP’s image quality. In the present study, all of the image post-processing was performed using a commercial image-processing tool (Adobe Photoshop CS3, version 10.0.1) by a single glaucoma-image-processing specialist (YKK). In detail, the histogram data of the downloaded high-resolution original ODPs (384 x 384 pixels) were evaluated to determine whether an image was over- or underexposed, flat (i.e., of little contrast), and the tonal range in which image adjustment was required. Then, using the Curves tool (specifically by clicking on the image Levels curve and dragging on it), the tonal ranges of an image were adjusted to improve its details and fine structures. Next, the visibility of retinal vessels or DH was enhanced by adjustment of the contrast and brightness between the blood vessels and background fundus. With the Selective color tool, red and yellow colors were completely replaced by green/blue (i.e., the blood vessel color was changed from red to bright red, and conversely, the background retinal color was changed from red-orange to light brown in order to maximize the visibility of hemorrhage). Then, with the Contrast/Brightness tool, the contrast and level were improved, and with the Smarten sharpen tool, the degree and range of the sharpness were increased (Fig 3). Finally, the ‘compensated high-resolution ODP’ could be obtained.

### Assessment of clinical implications of enhanced ODPs

**Fig 4:**
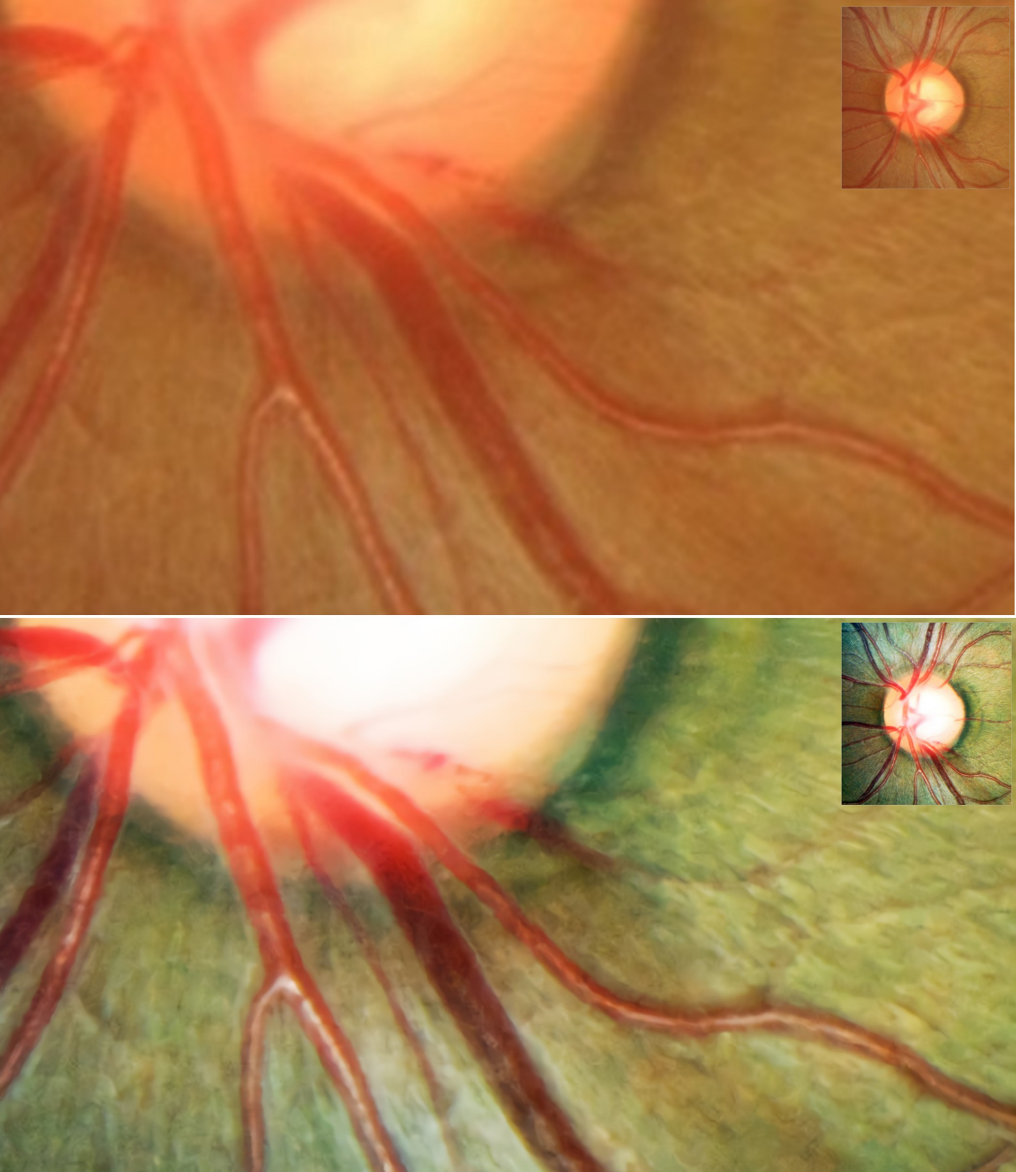
Representative optic-disc photography (ODP) of eye with optic disc hemorrhage (DH). (A) Magnified image of inferotemporal area in original high-resolution ODP, (B) Magnified image of inferotemporal area in deep-learning-based enhanced ODP. The enhanced ODP improved the color and spatial contrast between the DH and the background retinal color.

For the test, 50 high-resolution original ODPs and 50 paired SR-GAN-enhanced ODPs in two respective datasets were used. Three glaucoma specialists (AH, JL and KHP) independently evaluated the original ODPs of the test datasets and confirmed a total of 23 DHs in 23 original ODPs. Then, 12 general ophthalmologists were asked (1) to assess ODP image quality in 5 grades (excellent, good, fair, poor or bad), and (2) to note, for each of the original ODPs and enhanced ODPs separately at 1-month intervals, any abnormal findings including DH. In the process of the image quality grading, ‘excellent’ was defined as a clearly identified optic disc margin and distinct major vessel structures, while ‘bad’ was defined as unidentifiability of the optic disc margin. The ranges from good to fair quality and from fair to poor quality were determined subjectively by each ophthalmologist. The 5 grades were numbered between 1 (‘poor’) and 5 (‘excellent’); then, we performed a mean opinion score (MOS) test to compare the qualitative assessments in and among the image groups [17]. Fig 4 and S1 Fig. each compare an original ODP image with its enhanced version.

### Data analysis

All of the values are presented as means ± standard deviation. Paired *t* tests were used to determine the MOS differences between the two image types. The Mann-Whitney test was applied for comparison of the nonparametric data. The categorical data were analyzed by χ^2^ test, and a statistical analysis was performed using the SPSS statistical package (SPSS 22.0; Chicago, IL, USA.). A 2-sided *P*-value < 0.05 was considered to be statistically significant.

### Hardware specifications

CPU: Intel core i7-7700 3.60Hz x 8

GPU: TITAN X (Pascal) 12GB

RAM: 16GB

### Software specifications

Deep-learning libraries:

Tensorflow– 1.14.0 with cuda 10.0 and cudnn 7.6.3

Tensorflow Tensorlayer– 2.1.1

Python libraries (version—3.6)

Numpy– 1.16.4 for model loading and array processing

Scipy– 1.1.0 for image loading, resizing, saving

Scikit-image– 0.15.0 for image transformation (augmentation)

Matplotlib– 3.1.1 for plotting image

Easydict– 1.9 for dictionary values as attributes

Os–for filename load

Pickle–for vgg19 model loading

Random–for train data shuffle

Time–for calculating time for data loading, training, testing time

Optimizer:

Generator: AdamOptimizer–learning rate of 10e-4, beta1 of 0.9
Discriminator: AdamOptimizer–learning rate of 10e-4, beta1 of 0.9

## Results

### Training loss of modified SR-GAN

**Fig 5:**
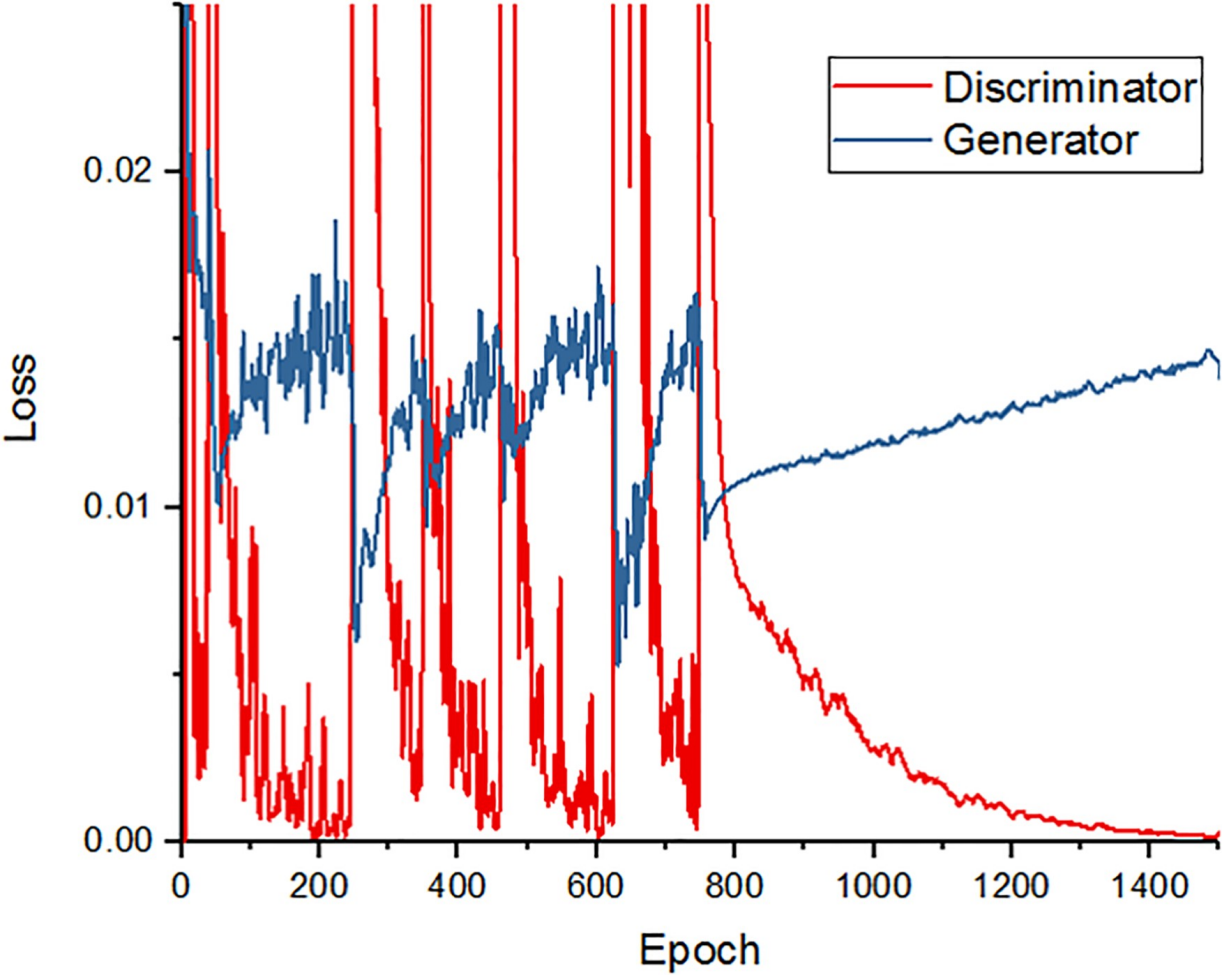
Training curve for deep-learning algorithm. The red line shows the accuracy of the discriminator over the training course, while the blue line represents the accuracy of the generator. As can be seen, after the 800th epoch, the discriminator’s loss decreases and the generator’s loss increases.

Fig 5 depicts the generator and discriminator loss over the course of the training set epochs. After 800 epochs, the loss of the discriminator decreased and that of the generator increased. Up to the 800^th^ epoch, the discriminator loss decreased and the generator loss increased for every 200 epochs, which is ideal for adversarial loss. After every 200 epochs, the generator loss abruptly decreased and the discriminator loss peaked. This is a characteristic of the Adam optimizer [18]. The value of the Adam optimizer soars when gradients are smaller and the whole denominator is smaller. Between the 800^th^ and 1400^th^ epochs, the loss values were stabilized. Accordingly, we trained our model to the 1500^th^ epoch.

### Performance of final network

We validated the enhanced ODPs according to Structural Similarity (SSIM) and Peak Signal-to-Noise Ratio (PSNR) and compared them with other machine-learning or state-of-the-art deep-learning methods [19]. SSIM is used to calculate the similarity between two images based on three measurements: luminance, contrast and structure.

$$SSIM(x,y)=\frac{\left(2\mu_{x}\mu_{y}+c_{1})(2\sigma_{xy}+c_{2}\right)}{\left({\mu_{x}}^{2}+{\mu_{y}}^{2}+c1)({\sigma_{x}}^{2}+{\sigma_{y}}^{2}+c_{2}\right)}$$ (5)

Here, *μ*_*x*_ and *μ*_*y*_ are the averages of x and y, *σ*_*x*_^2^, *σ*_*y*_^2^ are variances of x and y, and *σ*_*xy*_ is the covariance of *x* and *y*. *c*_1_ = (*k*_1_*L)*^2^, *c*_2_ = (*k*_2_*L)*^2^, where *L* is the dynamic range of pixel values, *k*_1_ = 0.001 and *k*_2_ = 0.003.

The optimization target of SR-GAN algorithms commonly is MSE minimization between the obtained and the targeted image. This is convenient, as minimizing the MSE also maximizes the PSNR, which is a measure commonly used to evaluate and compare SR algorithms.

$$MSE=\frac{1}{mn}\sum_{i=0}^{m-1}\sum_{j=0}^{n-1}{\left[I(i,j)-k(i,j)\right]}^{2}$$ (6)

$$PSNR={10\log}_{10}{\left(\frac{{MAX}_{I}}{MSE}\right)}^{2}$$ (7)

Here, *I*(*i*,*j*), *k*(*i*,*j*) describe the original image and the target image, respectively. MAX_I_ is the maximum pixel value of the image, and in the present case, the MAX_I_ value was 255. Also, the PSNR is calculated as the ratio between the maximum signal power and the noise power.

**Fig 6:**
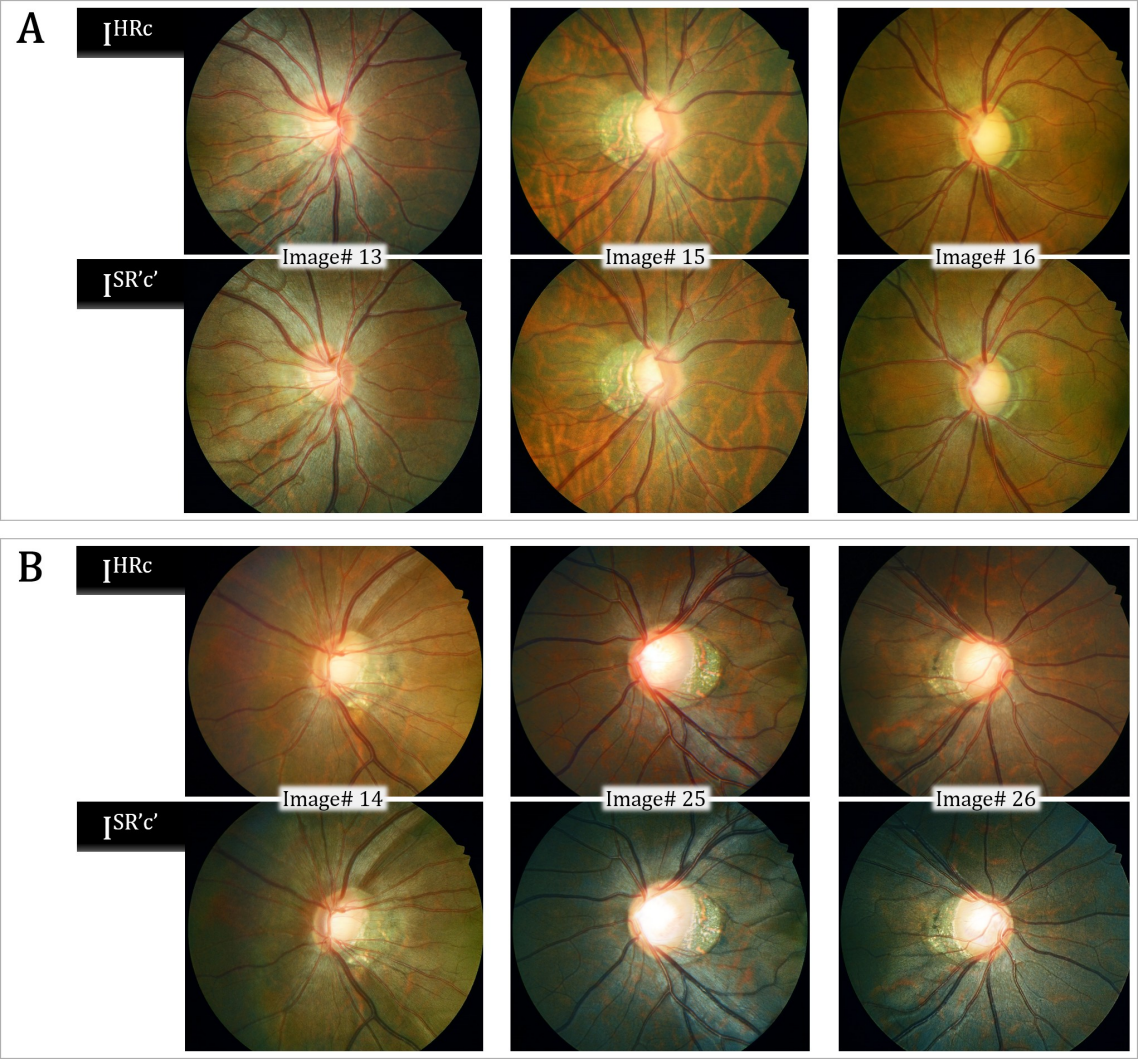
Validation results for representative test image sets. The Peak Signal-to-Noise Ratio (PSNR) and Structural Similarity (SSIM) values were higher in the upper 3 sets, and the obtained optic-disc photography (ODPs) were perceptually similar to the targeted ODPs (A). In the lower image sets, the change in background color caused relatively lower PSNR and SSIM values, even though those images were perceptually convincing (B).

The S1 and S2 Tables provide validation results for the representative six test image sets (all 384 x 384 pixel size). Both the mean SSIM and PSNR values were lower by our SR-GAN compared with other methods including Bicubic [20], NBSRF (Naive Bayes Super-Resolution Forest) [21], SR-RF (Super-Resolution Forests) [22], SRResNet (Super Resolution Residual Network) [10], and SRFBN (Feedback Network for Image Super-Resolution) [23]. Since our modified SR-GAN was designed to generate images to improve not only the resolution but also the color and spatial contrast, some of the enhanced images had greatly different color composition compared with the reference images (S2 Fig). By our modified SR-GAN method, the SSIM and PSNR values were higher in images #13, #15, and #16 than in images #14, #25, and #26. In images #13, #15, and #16, the obtained ODPs were similar to the targeted ODPs. In images #14, #25, and #26, however, the overall background color of the ODPs was transformed from red-orange to green. This might have caused the relatively lower SSIM and PSNR values, even though they were perceptually convincing images (Fig 6). The MOS test showed significant gains in perceptual quality using our modified SR-GAN compared with the Bicubic, NBSRF, SR-RF, SRResNet, and SRFBN methods (S3 Table).

### Clinical validation of enhanced ODP by MOS comparison

**Fig 7:**
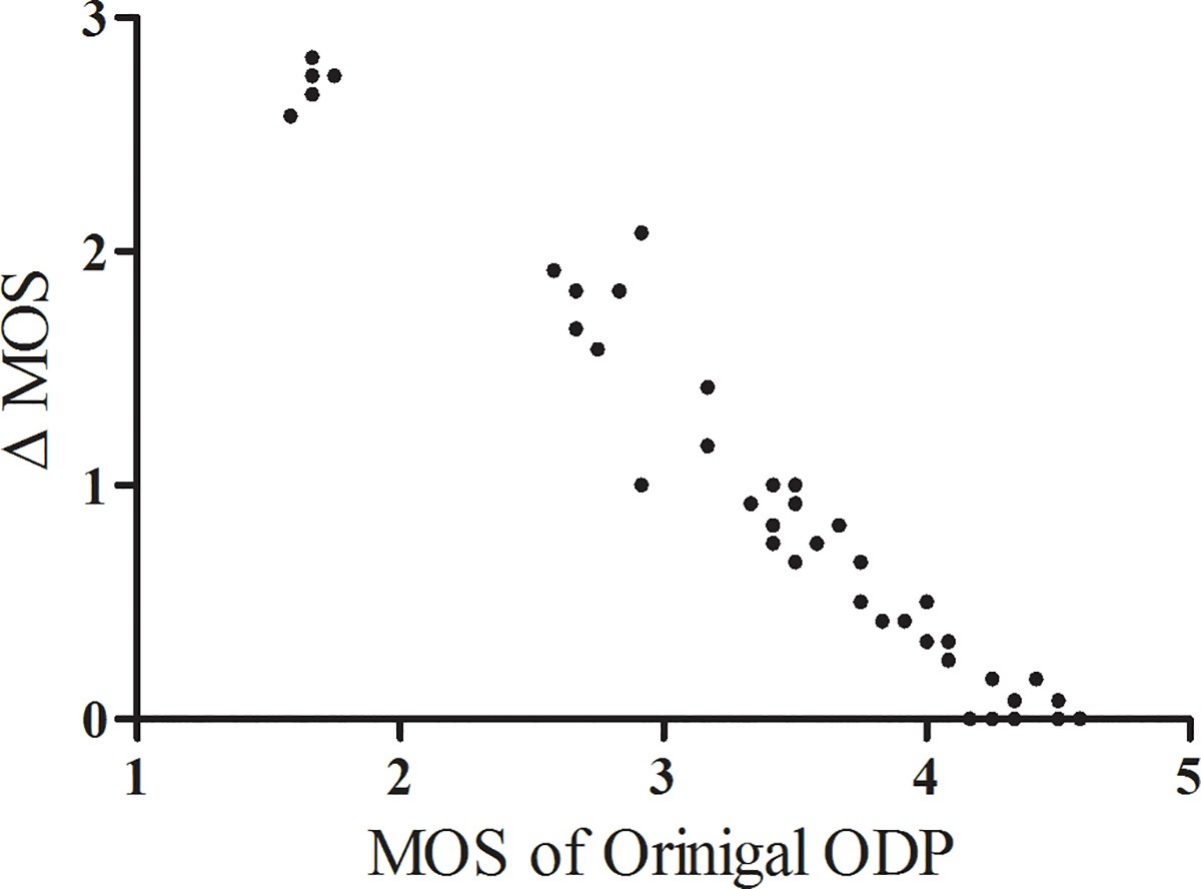
Scatter plot of delta mean opinion score (Δ MOS) against MOS of original optic-disc photography (ODP). The Δ MOS was calculated as the difference between the original and enhanced ODP scores. Note that the lower the original ODP image quality score, the larger the Δ MOS.

A total of 1200 responses comprising 12 ophthalmologists’ image quality assessments of 50 original and 50 enhanced ODPs were analyzed. The subjects’ demographic and ocular characteristics are provided in S4 Table. The image quality grades were numbered between 1 and 5 (higher scores indicating better quality), and all of the 50 enhanced ODPs were graded as either ‘excellent’ or ‘good.’ The MOS for the enhanced ODPs was significantly higher than that for the original ODPs (4.36 ± 0.38 *vs*. 3.51 ± 0.88, *P* < 0.001). The lower the original ODP image quality score, the larger the difference between the original and enhanced ODPs’ score (Fig 7).

### Comparison of DH-detection accuracy

The 12 ophthalmologists’ assessments of the 50 original ODPs and 50 enhanced ODPs were analyzed. The overall DH-detection accuracy was 76.3% with the original ODPs and 90.7% with the enhanced ODPs (*P* < 0.001). Among the misdiagnosed DHs, the rate of false-positive detection was 6.2 and 2.7% in the original and enhanced ODPs, respectively (*P* = 0.003). The rate of false-negative detection was 17.5 and 6.7% in the original and enhanced ODPs, respectively, and the difference was statistically significant (*P* < 0.001).

The group with the low original image quality (mean score < 3.0) showed a much improved DH-detection rate with the enhanced ODPs. The DH-detection accuracy differences between the original and enhanced ODPs were 29.5 ± 17.6 and 9.0 ± 12.8% in the low- and high-original-image-quality groups, respectively (*P* < 0.001).

## Discussion

We have presented herein a novel deep-learning approach to ODP enhancement that is capable of (1) 4-times up-scaling and (2) enhancement of anatomical details by means of contrast, color, and brightness improvement. We found that the resultant enhanced ODPs significantly improved general ophthalmologists’ accuracy of DH detection in glaucoma patients. The core novelty of our method lies in its clinical robustness in constructing image datasets. By applying a customized manual image post-processing algorithm to the training dataset, our network could improve both resolution and visibility of anatomical details, which compares favorably with other deep-learning approaches that focus only on resolution enhancement.

Recently, general image enhancement has achieved state-of-the-art performance, especially with the development of deep-learning techniques [24–26]. Dai et al. proposed a two-stage denoising method including fourth-order partial differential equations (PDEs) and a relaxed median filter for retinal image enhancement [27]. Bandara and Giragama applied a spatially adaptive contrast-enhancement technique for enhancement of fundus images [28]. However, some retinal pathologies (e.g., hemorrhages, microaneurysms, and drusen) are mostly only a few pixels wide, causing them to be easily confused with artifacts of noise. Thus, a fundus image enhancement method must be able to both suppress the undesired low-quality factors and preserve the pathological characteristics simultaneously, which requirement general enhancement techniques cannot satisfy [29]. Zhao et al. applied adversarial loss to blurry retinal images [30]. However, despite its computational efficiency, this method focuses only on generating photo-realistic images, ignoring lesions significant to clinical applications. Thus, in our study, we focused on designing an effective deep-learning model for robust images suitable to the diagnosis of ophthalmic pathologies.

In this study, 74% of the original ODPs were evaluated as ‘better than fair’ image quality sufficient for detection and diagnosis of pathologic change. However, on those original ODPs, a large percentage (142/600, 23.7%) of images was misdiagnosed by the ophthalmologists. This might have been owed to the fact that, even with high-resolution, good-quality ODPs, there is often limited detectability of small and indistinct pathologies due to insufficient time, fatigue, and/or lack of experience [7]. We demonstrated that by use of enhanced ODPs, detection accuracy for ONH pathology can be greatly improved.

We expect that this deep-learning approach for enhanced ODP will see wide application for accurate evaluation of ophthalmic pathologies and precision assessment of disease progression. Enhanced ODPs, for example, are expected to more clearly show vessel alterations or PPA changes associated with glaucomatous damage. Enhanced imaging enables ophthalmologists to zoom in on a suspect area and examine it in greater detail, without pixel loss. By application of this method to fundus photography, minute retinal hemorrhage or subtle enlargement of retinal nerve fiber layer defect can be more accurately and consistently detected.

**Fig 8:**
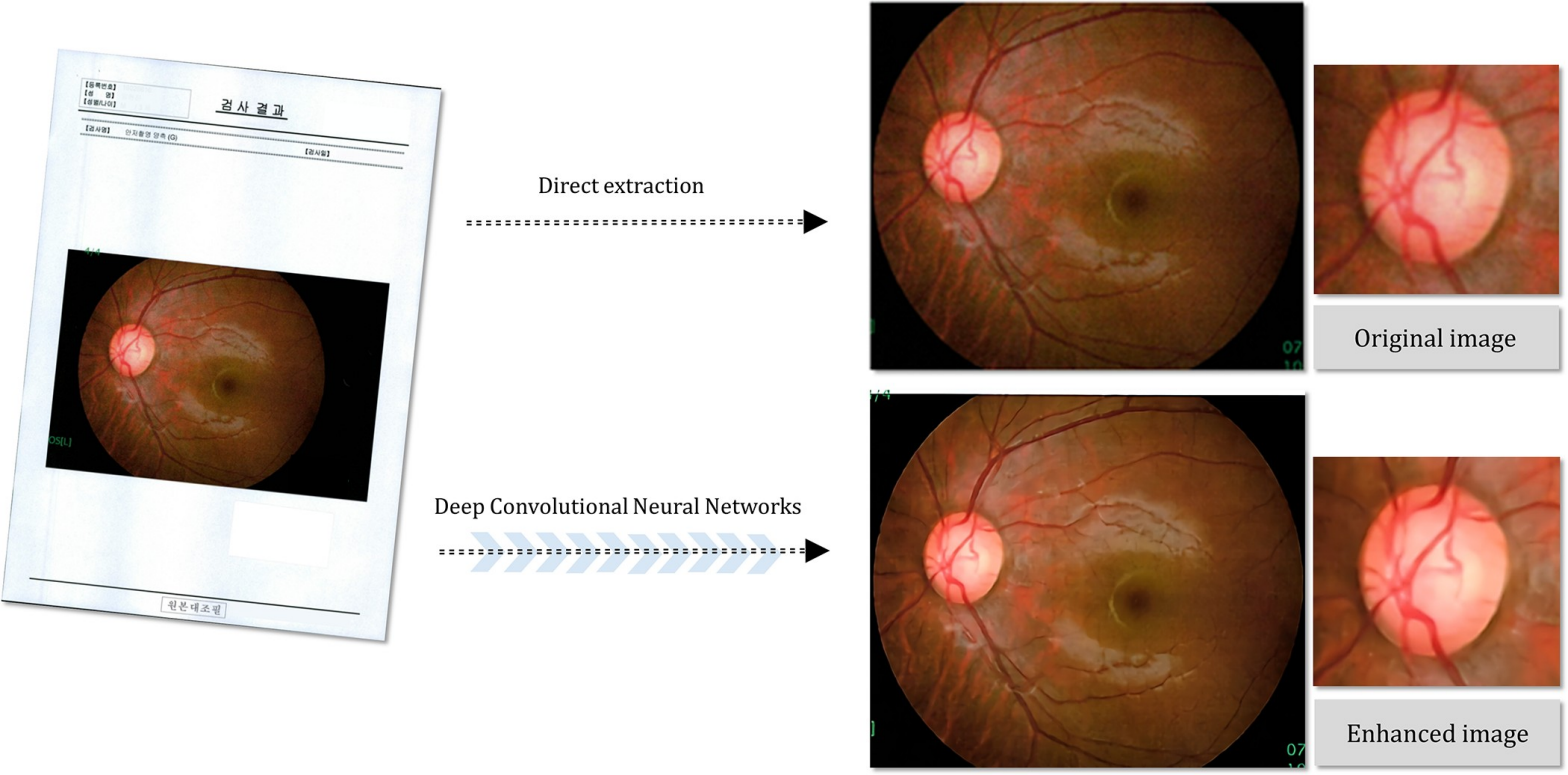
Example of fundus photography transferred from another institution as printed document. (A) Original document, (B) directly extracted fundus image, (C) magnified image of optic disc in original fundus photography, (D) deep-learning-based enhanced fundus photography, (E) magnified image of optic disc in enhanced fundus photography. The enhanced fundus photography improved the structural details of the optic disc, neuroretinal rim margin and vessel contours.

The clinical utility of deep-learning based image enhancement is particularly high in cases of low-quality images having low resolution and/or low contrast. In eyes with cataract or corneal opacity, enhanced ODPs can be used to improve overall image quality and the accuracy of glaucoma and retinal disease diagnostics. Moreover, images showing contrast loss due to poor focus, eye movement or insufficient illumination can be up-scaled. Regarding the examination results printed on paper in low resolution and transferred from other institutions, applying our deep-learning method for enhanced ODPs makes possible not only magnification of such images but also improvement of their structural details, which in turn allows for more meticulous evaluation (Fig 8). Additionally, there is growing interest in the value of using telemedicine for detection, following, and treatment of ophthalmic diseases [31, 32]. In cases of tele-ophthalmology requiring transmission of acquired low-resolution results [33, 34], transformation to enhanced images certainly can help to overcome hardware limitations, thereby enabling ophthalmologists to more closely analyze suspicious regions.

It has been reported that using GAN for super-resolution imaging can incur image artifacts in fine details [10]. Thus, two glaucoma specialists (AH and YKK) checked each image for the presence of artifacts, and could confirm that there were no such cases in our dataset. The underlying reason for this difference in artifact occurrence rate is not yet clear. We speculate that previous GANs are more vulnerable to artifacts because they utilize a variety of images as a training dataset, as compared with ours, which consists only of ODPs. However, since artifacts in medical applications may affect diagnosis or management of patients, caution needs to be exercised in any attempts to utilize our network for other image types.

Single-image SR via deep learning recently has attracted significant research attention. In the present study, a modified SR-GAN consisting of a GAN and a pre-trained VGG19 network was adopted for image training. A network trained for image classification (like VGG) stores, in its feature maps, detailed information on the appearance of common objects, thus enabling an up-scaled image to be made up, to the extent possible, of objects resembling real-world ones. A GAN also has additional merits including non-dependence on prior-knowledge, the lack of any need to design hand-engineered features, and high effectiveness in capturing image structures. Such underlying advantages render SR-GAN a robust platform allowing for multiple applications to be followed once well-trained SR artificial intelligence is established. In future studies, we will explore this modified SR-GAN’s results for different datasets.

It is known that deep learning generally requires a large dataset for the training phase [16]. In the current study however, we demonstrated the ability to obtain clinically meaningful results with only 48 pairs of datasets. We carefully modeled both the image degradation process for generation of low-resolution ODPs and the image customization process for production of compensated high-resolution ODPs; in this way, we eliminated the need for complicated alignment of high- and low-resolution pairs. These steps simplified data processing and improved the modified GAN’s robustness. We believe that this example-based method using standardized low- and high-resolution image pairs can maximize the time efficiency of the training process.

The present study’s findings must be interpreted in light of its limitations. First, numerical evaluation of enhanced image quality was unsuitable for some of our dataset [35]. Different metrics, such as PSNR, SSIM, and multi-scale SSIM, are widely used for quantitative assessment of image restoration quality [36]. These metrics measure reconstructed image quality with respect to the reference or ground-truth image. Some of our enhanced ODPs had greatly different color composition compared with the reference image, due to the fact that we had used compensated ODPs with color-contrast customization in the training process [37]. With such alterations in color composition, direct comparison by numerical evaluation with other deep-learning methods that focus only on resolution improvement would be inappropriate [37]. Furthermore, none of these metrics are known to be well matched with human visual responses to image quality [16]. For these reasons, we focused on the clinical implications of the use of enhanced ODPs for diagnosis of optic disc pathology. Further numerical evaluation of enhanced ophthalmic image quality with reasonable metrics should be carried out in future studies. Second, we applied a customized image post-processing algorithm for optimization of both the color and spatial contrast of each ODP. This detailed manual adjustment was applied differently according to each ODP’s image quality. Although this variability in the image-processing procedure may incur reproducibility issues, we believe that the core novelty of our method lies in the customization of image-processing procedures. In real-world clinical practice, ODPs can have several different limitations other than insufficient resolution, such as low color and spatial contrast. Based on the customized image compensation process to optimize the visibility of ophthalmic pathology, we enabled GAN to generate enhanced ODPs with both higher resolution and improved anatomical details. However, different image post-processing methods or training strategies might manifest different results. Third, the clinical implications of enhanced ODP were not evaluated for other optic-disc characteristics such as neuroretinal rim contours. This was due to the fact that the image-compensation process of the present study was mainly focused on the enhancement of the visibility of the optic disc margin and surrounding retinal vessels, not on the rim contours. Therefore, further research is certainly needed to determine the usefulness of deep-learning-based enhanced ODP in glaucoma diagnostics. Fourth, our meaningful training results were based on relatively little data. Further studies will validate this algorithm using a larger dataset.

The current study demonstrated that deep learning can be applied to create an algorithm that is capable of producing enhanced ophthalmic images that are 4-times up-scaled and improved in their structural details. The enhanced ODPs thereby obtained significantly increased the detection accuracy of optic disc pathology. Further studies exploring the usefulness of this algorithm’s deployment in different clinical settings are warranted.

## Supporting information

S1 Fig Representative optic-disc photography (ODP) of eye with tilted optic disc and parapapillary chorioretinal atrophy (PPA). (A) Original high-resolution ODP, (B) deep-learning-based enhanced ODP, (C) magnified image of inferotemporal area in original high-resolution ODP, (D) magnified image of inferotemporal area in deep-learning-based enhanced ODP. The enhanced ODP enabled image magnification without pixel loss; thus, the details of the disc margin, PPA border, and small-caliber vessels could be clearly shown.
(TIF)

S2 Fig Representative optic-disc photography (ODP) reconstruction results and corresponding reference images. From left to right: original high-resolution ODP, bicubic interpolation, SR-RF (Super-Resolution Forests), NBSRF (Naive Bayes Super-Resolution Forest), SRFBN (Feedback Network for Image Super-Resolution), SRResNet (Super Resolution Residual Network), and our SR-GAN (super-resolution generative adversarial network) [x4 up-scaling]. (A) SDP of left eye of patient diagnosed with glaucoma suspect, (B) SDP of left eye of glaucoma patient with tilted optic disc and parapapillary chorioretinal atrophy, and (C) SDP of right eye of glaucoma patient with inferotemporal optic disc hemorrhage.
(TIF)

S1 Table. Comparison of SSIM index values for representative test images (DOCX)

S2 Table. Comparison of PSNR values for representative test image sets (DOCX)

S3 Table Mean opinion score (MOS) test results for representative test image sets. (DOCX)

S4 Table Demographic and clinical characteristics of study subjects. (DOCX)
