# Supplementary figures and images for: Deep-learning-based enhanced optic-disc photography
Source: PLoS One. 2020 Oct 1;15(10):e0239913. doi: 10.1371/journal.pone.0239913 (PMC7529226; doi:10.1371/journal.pone.0239913)

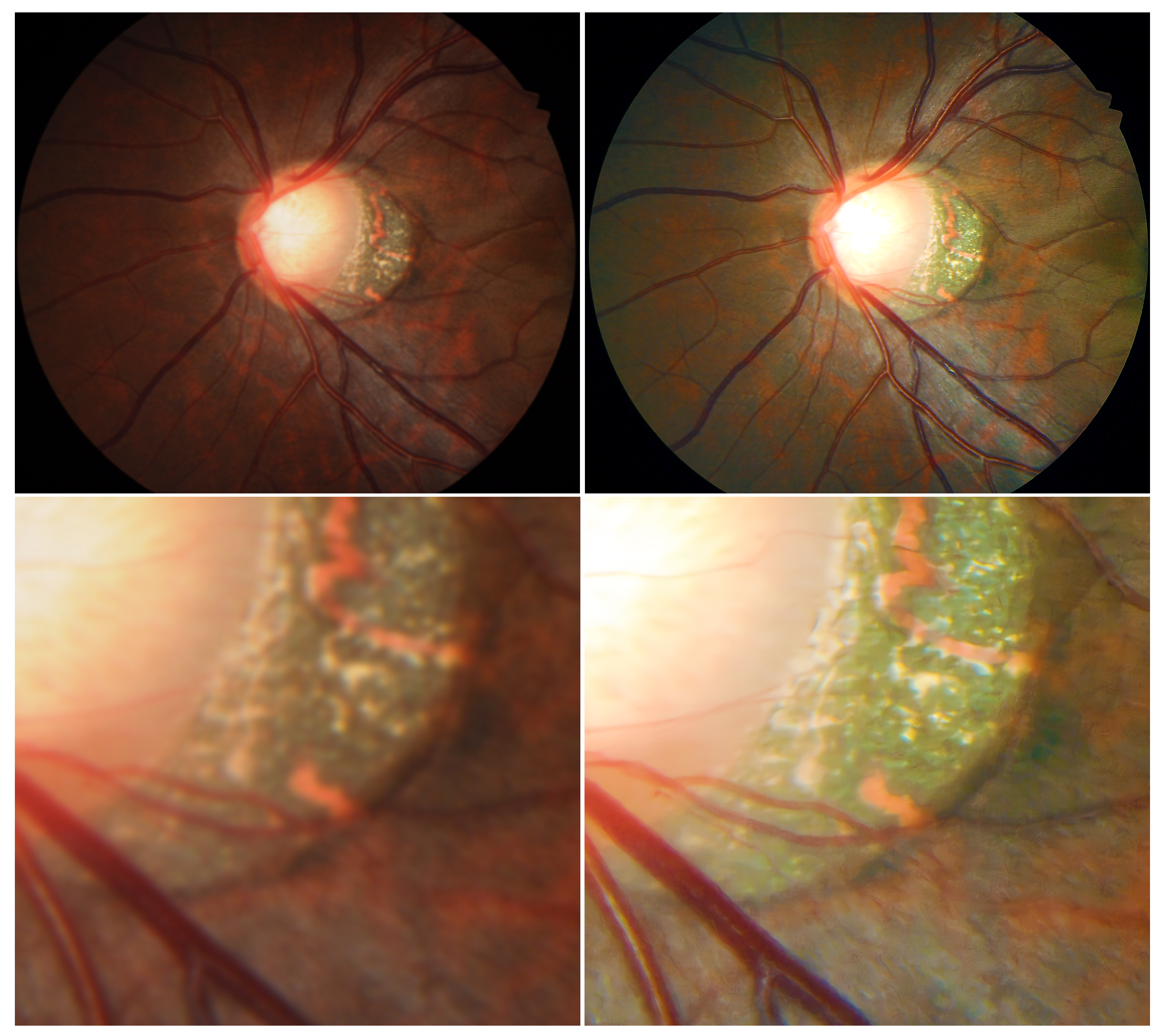

Supplement: S1 Fig — (A) Original high-resolution ODP, (B) deep-learning-based enhanced ODP, (C) magnified image of inferotemporal area in original high-resolution ODP, (D) magnified image of inferotemporal area in deep-learning-based enhanced ODP. The enhanced ODP enabled image magnification without pixel loss; thus, the details of the disc margin, PPA border, and small-caliber vessels could be clearly shown. (TIF) [file pone.0239913.s001.tif]

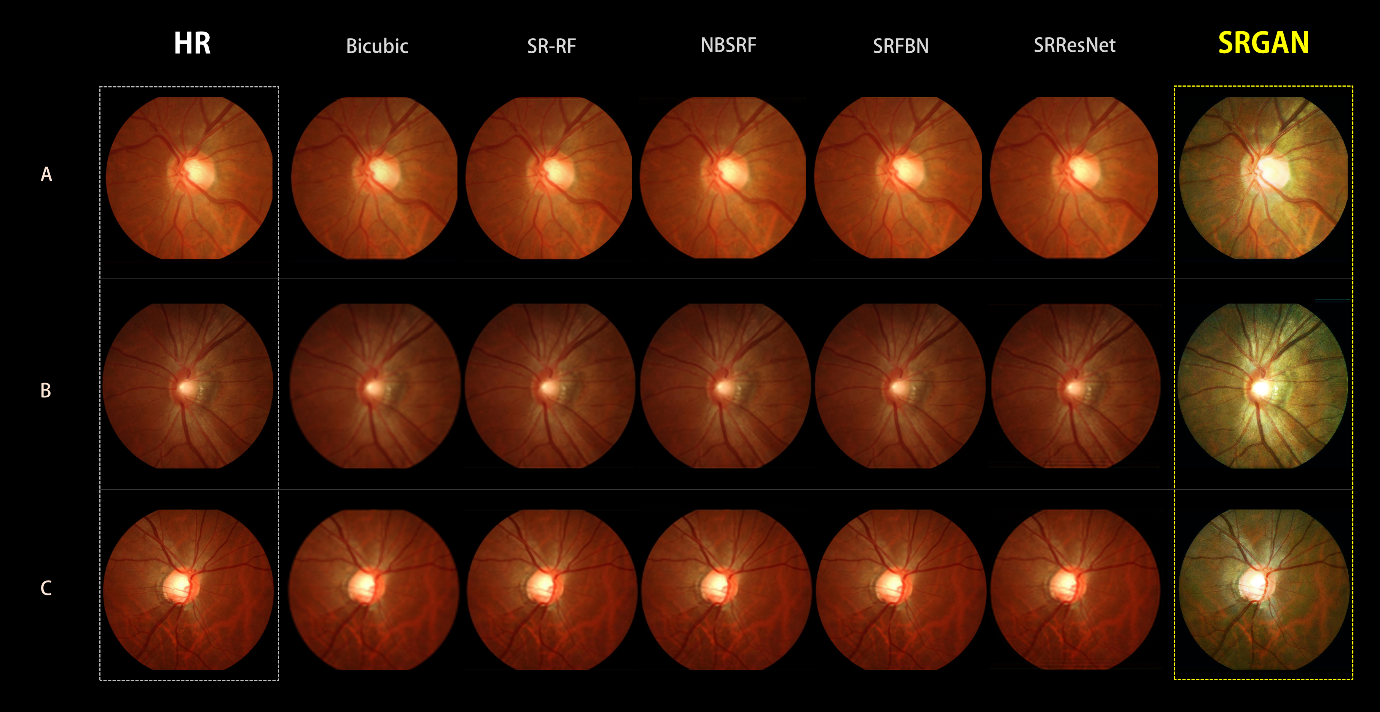

Supplement: S2 Fig — From left to right: original high-resolution ODP, bicubic interpolation, SR-RF (Super-Resolution Forests), NBSRF (Naive Bayes Super-Resolution Forest), SRFBN (Feedback Network for Image Super-Resolution), SRResNet (Super Resolution Residual Network), and our SR-GAN (super-resolution generative adversarial network) [x4 up-scaling]. (A) SDP of left eye of patient diagnosed with glaucoma suspect, (B) SDP of left eye of glaucoma patient with tilted optic disc and parapapillary chorioretinal atrophy, and (C) SDP of right eye of glaucoma patient with inferotemporal optic disc hemorrhage. (TIF) [file pone.0239913.s002.tif]
