# Supplementary material for: Deep-learning-based enhanced optic-disc photography
Source: PLoS One. 2020 Oct 1;15(10):e0239913. doi: 10.1371/journal.pone.0239913 (PMC7529226; doi:10.1371/journal.pone.0239913)
Supplement: S1 Table. Comparison of SSIM index values for representative test images — (DOCX) [file pone.0239913.s003.docx]

**S1 Table. Comparison of the SSIM index values for the representative test images.**

| **SSIM** | **# 13** | **# 15** | **# 16** | **# 14** | **# 25** | **# 26** | **mean** |
| --- | --- | --- | --- | --- | --- | --- | --- |
| Bicubic | 0.89 | 0.90 | 0.92 | 0.92 | 0.90 | 0.91 | 0.91 |
| SRRF | 0.90 | 0.90 | 0.93 | 0.91 | 0.90 | 0.90 | 0.91 |
| NBSRF | 0.92 | 0.93 | 0.95 | 0.93 | 0.92 | 0.93 | 0.93 |
| SRFBN | 0.92 | 0.93 | 0.95 | 0.93 | 0.92 | 0.93 | 0.93 |
| SRRESNET | 0.90 | 0.91 | 0.93 | 0.91 | 0.90 | 0.91 | 0.91 |
| Modified SR-GAN | 0.91 | 0.97 | 0.95 | 0.78 | 0.43 | 0.43 | 0.75 |
| SSIM, structural similarity; SRRF, Super-Resolution Forests; NBSRF, Naive Bayes Super-Resolution Forest; SRFBN, Feedback Network for Image Super-Resolution; SRRESNET, Super-Resolution Residual Network; SR-GAN, Super-Resolution Generative Adversarial Network | | | | | | | |
|  |  |  |  |  |  |  |  |
|  |  |  |  |  |  |  |  |
