# Supplementary material for: Deep-learning-based enhanced optic-disc photography
Source: PLoS One. 2020 Oct 1;15(10):e0239913. doi: 10.1371/journal.pone.0239913 (PMC7529226; doi:10.1371/journal.pone.0239913)
Supplement: S2 Table. Comparison of PSNR values for representative test image sets — (DOCX) [file pone.0239913.s004.docx]

**S2 Table. Comparison of the PSNR values for the representative test image sets.**

| **PSNR (dB)** | **# 13** | **# 15** | **# 16** | **# 14** | **# 25** | **# 26** | **mean** |
| --- | --- | --- | --- | --- | --- | --- | --- |
| Bicubic | 36.17 | 36.13 | 38.06 | 37.60 | 36.46 | 37.47 | 36.98 |
| SRRF | 33.20 | 32.22 | 33.49 | 34.41 | 34.77 | 34.97 | 33.84 |
| NBSRF | 38.36 | 38.72 | 40.81 | 39.61 | 37.70 | 39.02 | 39.04 |
| SRFBN | 38.72 | 38.98 | 41.33 | 39.99 | 37.90 | 39.34 | 39.38 |
| SRRESNET | 36.98 | 37.13 | 38.72 | 88.20 | 36.79 | 37.80 | 37.60 |
| Modified SR-GAN | 27.95 | 29.81 | 28.86 | 20.05 | 21.69 | 21.71 | 25.01 |
| PSNR, peak signal-to-noise ratio; SRRF, Super-Resolution Forests; NBSRF, Naive Bayes Super-Resolution Forest; SRFBN, Feedback Network for Image Super-Resolution; SRRESNET, Super-Resolution Residual Network; SR-GAN, Super-Resolution Generative Adversarial Network | | | | | | | |
|  |  |  |  |  |  |  |  |
|  |  |  |  |  |  |  |  |
