# Supplementary material for: Deep-learning-based enhanced optic-disc photography
Source: PLoS One. 2020 Oct 1;15(10):e0239913. doi: 10.1371/journal.pone.0239913 (PMC7529226; doi:10.1371/journal.pone.0239913)
Supplement: S3 Table — (DOCX) [file pone.0239913.s005.docx]

**S3 Table. Mean opinion score (MOS) test results for the representative test image sets.**

| **MOS** | **# 13** | **# 15** | **# 16** | **# 14** | **# 25** | **# 26** | **mean** |
| --- | --- | --- | --- | --- | --- | --- | --- |
| Bicubic | 2.87 | 2.75 | 2.95 | 3.05 | 2.84 | 3.08 | 2.92 |
| SRRF | 2.96 | 3.12 | 3.56 | 3.00 | 2.88 | 2.94 | 3.08 |
| NBSRF | 2.98 | 3.15 | 3.64 | 3.25 | 2.86 | 2.92 | 3.13 |
| SRFBN | 3.87 | 4.05 | 4.12 | 3.91 | 3.75 | 3.80 | 4.06 |
| SRRESNET | 3.73 | 3.96 | 4.02 | 3.88 | 3.74 | 3.61 | 3.82 |
| Modified SR-GAN | 4.25 | 4.46 | 4.52 | 4.11 | 4.28 | 4.33 | 4.33 |
| MOS, mean opinion score; SRRF, Super-Resolution Forests; NBSRF, Naive Bayes Super-Resolution Forest; SRFBN, Feedback Network for Image Super-Resolution; SRRESNET, Super-Resolution Residual Network; SR-GAN, Super-Resolution Generative Adversarial Network | | | | | | | |
|  |  |  |  |  |  |  |  |
|  |  |  |  |  |  |  |  |
