# Supplementary material for: Deep-learning-based enhanced optic-disc photography
Source: PLoS One. 2020 Oct 1;15(10):e0239913. doi: 10.1371/journal.pone.0239913 (PMC7529226; doi:10.1371/journal.pone.0239913)
Supplement: S4 Table — (DOCX) [file pone.0239913.s006.docx]

| **Table S4. Demographic and Clinical Characteristics of Study Subjects (N = 98)** | |
| --- | --- |
| Characteristics | Values |
| Age (yrs) | 57.5 ± 11.9 |
| Male, n (%) | 46 (46.9) |
| Spherical equivalent (D) | -1.4 ± 3.0 |
| IOP (mmHg) | 14.7 ± 3.4 |
| CCT (μm) | 541.6 ± 32.4 |
| Axial length (mm) | 24.5 ± 1.75 |
| VF MD (decibels) | -4.5 ± 8.6 |
| Values are mean ± standard deviation.  D, diopters; IOP, intraocular pressure; CCT, central corneal thickness; VF, visual field; MD, mean deviation. | |
